# Effect of Combined Antihypertensive and Lipid-Lowering Therapies on Cognitive Function: A New Treatment Strategy?

**DOI:** 10.1155/2020/1484357

**Published:** 2020-04-14

**Authors:** Ze-Min Kuang

**Affiliations:** Department of Hypertension, Beijing Anzhen Hospital of Capital Medical University, Beijing 100029, China

## Abstract

Risk factors for cardiovascular disease such as hypertension and hyperlipidemia are associated with cognitive decline. However, there is still no clear evidence that the use of antihypertensive or lipid-lowering therapy can prevent or delay cognitive decline or development of dementia. To provide a reference for clinical treatment, we analyzed the potential mechanisms of cognitive dysfunction induced by hypertension and hyperlipidemia, the clinical research and controversy of antihypertensive and lipid-lowering therapies on cognitive function, and the clinical value of combined antihypertensive and lipid-lowering therapy. It is currently believed that hypertension and elevated blood cholesterol levels in middle-aged people may be related to cognitive impairment or dementia in the elderly. Some studies suggest that intensive antihypertensive or lipid-lowering therapies are better than standard antihypertensive or lipid-lowering therapy, yet further tests are needed to confirm their effects on cognitive function. Actively controlling potential risk factors from middle age may be important for Alzheimer's disease (AD) prevention.

## 1. Introduction

Cardiovascular risk factors including hypertension and hyperlipidemia are associated with cognitive decline and dysfunction [1, 2]. However, it is still controversial whether controlling cardiovascular risk factors can prevent cognitive decline or dementia, despite some speculations that altering blood pressure, blood lipid levels, and other cardiovascular risk factors will reduce the incidence of dementia in these populations [3]. However, there is still a lack of long-term and large-scale clinical randomized controlled trials to explore the relationship between blood pressure, lipid levels, and cognitive impairment. In this review we will focus on whether antihypertensive, lipid-lowering, or combined therapy can prevent or delay the occurrence of cognitive impairment.

It is well-accepted that elevated middle-aged blood pressure is associated with cognitive decline [4, 5], but it is not clear whether antihypertensive therapy can prevent cognitive decline. On the other hand, hyperlipidemia is also associated with cognitive impairment, but the results are still controversial. Studies have shown that the apolipoprotein E (APOE) *ε*4 allele and elevated total cholesterol and systolic blood pressure (SBP) in middle age are independent risk factors for AD, and the combined risk of elevated total cholesterol and elevated blood pressure seems to be higher than that of the APOE*ε*4 allele [6]. We hope to explore whether combined antihypertensive and lipid-lowering drugs can play a synergistic role and further prevent or slow down the decline of cognitive function or development of dementia based on the existing literature.

## 2. Effect of Hypertension and Antihypertensive Therapy on Cognitive Function

### 2.1. Potential Mechanism of Hypertension on Cognitive Dysfunction

**Figure 1:**
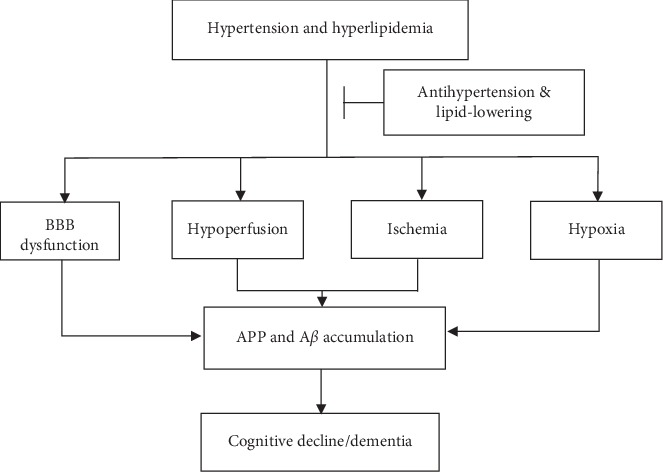
Potential mechanism of hypertension and hyperlipidemia on cognitive decline. BBB: blood-brain barrier; APP: amyloid precursor protein; A*β*: beta-amyloid peptides.

Hypertension causes changes in the vascular walls which can lead to hypoperfusion, ischemia and hypoxia, and cognition decline [7]. Previous studies demonstrated that cerebral ischemia led to the accumulation of amyloid precursor protein (APP) and beta-amyloid peptides (A*β*), in addition to stimulating the expression of presenilin, a protein involved in A*β* synthesis [8]. Hypertension may also lead to dysfunction in the blood-brain barrier, worsen vascular endothelial injury, change cerebral white matter lesion volume, and decrease total brain volume including hippocampal volume and angiosclerosis, which can damage cognitive function [9–11]. Elevated levels of Alzheimer-associated neuronal thread protein (AD7c-NTP) were found in the urine of elderly hypertensive patients with lower cognitive function, and insulin resistance may be involved in the process as well [12]. In rat hypertension model, Okura and Higaki demonstrated that nicotinic acetylcholine receptors were related to rats' learning and memory ability, and hypertension caused the decline in number of neurons. This study provided the experimental evidence for the effects of hypertension on the cognitive impairments [13] (see Figure 1).

### 2.2. Controversy: Can Antihypertensive Therapy Affect Cognitive Impairment?

In 2011, the American Heart Association (AHA)/American Stroke Association (ASA) made the following recommendations for blood pressure management and cognitive function protection in high-risk populations of dementia based on six large clinical randomized trials and five meta-analyses [14]: (1) in people with a history of stroke, antihypertensive therapy can effectively prevent poststroke dementia (Class I, Level of Evidence B); (2) in middle and younger ages, antihypertensive therapy can prevent dementia (Class IIA recommendation, Level of Evidence B); (3) in the population over age 80, the effectiveness of antihypertensive therapy for the prevention of dementia is not clear (Class IIB, Level of Evidence B); and (4) antihypertensive therapy is necessary in people with cognitive impairment caused by cardiovascular disease (Class I recommendation, Level of Evidence A). These statements only provide general recommendations of the relationship between antihypertensive therapy and protection of cognitive function. They do not give further advice on drug selection, timing of treatment, or blood pressure control and are, therefore, not practical for clinicians.

Hypertension has recently been recognized as a risk factor of cognitive decline/dementia [15, 16]. It is currently believed that middle-aged hypertension is associated with cognitive impairment in the elderly and that elderly hypotension is associated with an increased risk of cognitive impairment [17–19]. The duration of hypertension and the trajectory of blood pressure (BP) over time seemed to significantly affect the risk of cognitive decline [20, 21]. However, another study showed that duration of arterial hypertension had no significant adverse effect on the Mini-Mental State Examination (MMSE), but age had a significant effect on cognitive impairment [22]. Early-onset hypertension in childhood and adolescence and a duration of hypertension over 25 years significantly increased the risk of dementia, with an increase in middle-aged BP combined with lower diastolic hypertension in the later years [20, 21, 23].

The 2018 European Society of Cardiology (ESC)/European Society of Hypertension (ESH) Guidelines for the management of arterial hypertension have little evidence of the beneficial effects of antihypertensive therapy on cognitive decline [24]. There is an urgent need to conduct trials to better determine if antihypertensive therapy can prevent cognitive decline or delay the potential effects of dementia when cognitive dysfunction already exists. However, an article published in PLoS Medicine in 2018 did not show any benefit from antihypertensive therapy in patients with mild-to-moderate AD [25]. Recent studies have demonstrated that short-term mild hypertension may be a protective factor for mild cognitive impairment in people over the age of 70 [26].

Therefore, it makes us think whether antihypertensive therapy can prevent or delay the occurrence of cognitive impairment. A previous longitudinal study found that subjects who developed dementia at age 79 to 85 had significantly higher blood pressures 15 years earlier [27]. Dementia may be the result of chronic and long-term high blood pressure, but not a transient process. Thereafter, the Honolulu-Asia aging study discovered that untreated hypertension was significantly associated with hippocampal atrophy, midlife cognitive decline, AD, and vascular dementia [28]. Interestingly, diastolic hypertension was more strongly associated with hippocampal atrophy than systolic hypertension [28]. In the HYVET study, a double-blind, placebo-controlled trial of antihypertensives in patients aged at least 80 years, it was demonstrated that a wider pulse pressure may indicate an increased risk for dementia, and it was also found that active treatment may change the relationship between diastolic hypertension and dementia [29]. Amazingly, a clinical trial showed that treating isolated systolic hypertension reduced Alzheimer's dementia by 50% [30]. Therefore, active control of systolic hypertension from middle age is important for preventing AD [31].

Different types of antihypertensive drugs have different cognitive improvements. A network meta-analysis showed that antihypertensive treatment attenuates cognitive decline and prevents dementia, and indicated that these effects may differ from drug classes, among which angiotensin receptor blockers (ARBs) were the most effective [32]. Similarly, some researchers have shown that ARBs may have particular advantages [33]. Some researchers found that both calcium channel blockers (CCBs) and ARBs are independently associated with a decreased risk of dementia in older people [34]. In addition, antihypertensive adherence is an important factor impacting the odds of dementia, which is three times greater for those with moderate antihypertensive adherence compared to those with near perfect adherence [35].

### 2.3. Blood Pressure Control Prevents or Delays Cognitive Impairment: Intensive Treatment versus Conventional Treatment

Clinical research published in JAMA in 2019 holds the view that intensive systolic blood pressure control can prevent cognitive impairment [36]. The idea of reducing systolic blood pressure to 150 mmHg or lower has been controversial. One risk often mentioned is the possibility that hypotension and cerebral hypoperfusion have a negative impact on the brain. However, this trial did not observe this negative effect; specifically, these results indicated that after a 3.34-year median intervention period, blood pressure (BP) control did not impair cognitive perception during a total median follow-up of 5.11 years. In addition, there are some indications that may be beneficial to strengthen the control of BP. To the best of our knowledge, this is the first trial to demonstrate that an intervention can significantly reduce the incidence of mild cognitive impairment (MCI), which is a recognized risk factor for dementia. However, caution should be exercised in interpreting this result because MCI was not the primary cognitive outcome of the trial, and it is not clear what this effect might mean for the long-term morbidity of dementia. Although MCI increases the risk of dementia progression, this progression is uncertain, and it is possible for cognition to return to normal. A multicenter study in the United States showed a significant cognitive decline after 40 months of intensive BP control in patients with type 2 diabetes [37].

Information on all the clinical trials mentioned above, namely, research design, age at baseline, intervention, duration/follow-up, main results, and the number of participants, has been summarized in Table 1.

## 3. Effect of Hyperlipidemia and Statin Lipid-Lowering Therapy on Cognitive Function

### 3.1. Potential Mechanism of Hyperlipidemia on Cognitive Function

Previous studies using animal models showed that hypercholesterolemia is associated with increased A*β* peptide deposition, in addition to increased neurofibrillary tangles formation, neuroinflammation, dysfunction of cholinergic neurons, and cerebral microhemorrhages, which may contribute to cognitive decline [38, 39]. In addition, studies have shown that elevated circulating cholesterol levels are capable of compromising the integrity of the blood-brain barrier [40]. High-density lipoprotein (HDL) is an important carrier of cerebral cholesterol; low levels of HDL may cause increased sediment of A*β* proteins and induce inflammation [41]. In cases of hyperlipidemia, free-radical scavenger activity declines, which causes a large accumulation of lipid peroxide, accelerates the development and progression of atherosclerosis, and reduces cerebral blood flow, resulting in cerebral ischemia and hypoxia, brain tissue damage, and, ultimately, cognitive impairment [42] (see Figure 1).

### 3.2. Disputes: Can Statin Lipid-Lowering Therapy Affect Cognitive Impairment/Dementia?

The relationship between blood lipids and cognition is very complex and controversial. Elevated blood cholesterol in middle-aged patients increases the risk of AD and vascular dementia and emphasizes the need to resolve the risk factors of dementia before middle age or the onset of potential diseases or symptoms [43–45]. However, a longitudinal Japanese study with a 3-year follow-up showed that the presence of dyslipidemia and higher educational levels are protective factors of cognitive decline [46]. Meanwhile some observational studies have found that the use of statins promotes cognitive decline [47], and in 2012 a review published by the Food and Drug Administration (FDA) showed that there is some limited evidence that statin use can lead to cognitive impairment [48, 49]. However, contrary to these observational studies, meta-analysis of randomized trials did not reveal any adverse effects of statins on cognition [50].

The midlife measures of total cholesterol were significant predictors of cognitive impairment [51], especially the association between increased HDL cholesterol levels and better cognitive performance [52–54]. In contrast, high LDL levels were associated with lower risk of cognitive impairment in the oldest elderly (aged 80 and older), but not in the younger elderly (aged 65 to 79 years) [53, 55]. Improved cognition was associated with lower triglyceride only in males [56]. However, a recent study showed that high total serum cholesterol in later life compared to midlife was not associated with any form of dementia or cognitive decline [45].

A number of observational studies have found an association between the control of dyslipidemia and the reduction of dementia and/or cognitive decline risk [57–59]. Therefore, the 2019 World Health Organization (WHO) guidelines recommend indirect evidence that controlling dyslipidemia in middle age can help reduce the risk of cognitive decline and/or dementia. The Rotterdam study in the general population found that the use of statins was associated with a lower risk of AD compared with no usage of cholesterol-lowering drugs [60]. A recent article examined the association between statin use and changes in memory and global cognition in community-dwelling elderly Australians (aged 70 to 90 years) over 6 years and brain volumes over 2 years. They found no difference in the rate of decline in brain volume, memory, or global cognition between statin users and never users [61]. Interestingly, they also found that statin use was associated with attenuated specific memory decline in those with heart disease and APOE*ε*4 carriage. Similarly, the use of statins may benefit all AD patients with potentially greater therapeutic efficacy in those homozygous for APOEε4 [62].

However, there are also some studies that oppose an association between statin therapy and a decreased risk of dementia [35, 63–65]. A review showed that there was no evidence that lipid-lowering therapy can improve the cognition of patients with dementia and vascular dementia and there was a lack of data for middle-aged patients [66]. Although evidence of randomized controlled trial to challenge clinical decisions is lacking, it offers multiple ongoing and future research opportunities.

### 3.3. Lipid-Lowering Therapy Prevents or Delays Cognitive Impairment: Intensive Treatment versus Conventional Treatment

The Pilot Prevention of Decline in Cognition after Stroke Trial (PODCAST) randomized controlled trial showed that intensive lipid-lowering therapy (target LDL-c < 1.3 mmol/l) was feasible and safe in patients with recent stroke, nondementia, and total cholesterol 3.0–8.0 mmol/l, but it did not change cognition for two years [67]. There is also a study that showed that intensive lipid-lowering treatment can modify the deterioration of neurocognitive function and the loss of volume in certain cerebral areas in older patients with arterial fibrillation [68]. Therefore, further trials are needed to confirm the effects of intensive lipid-lowering on cognitive function.

Information on all the clinical trials mentioned above, namely, research design, age at baseline, intervention, duration/follow-up, main results, and the number of participants, has been summarized in Table 2.

## 4. Combined Antihypertensive and Lipid-Lowering Therapies: A New Treatment Strategy on Cognitive Function?

### 4.1. Mechanism of Antihypertensive and Lipid-Lowering Therapy on Cognition

Although there is no clear mechanism for the combination of antihypertensive and lipid-lowering therapy on cognitive function, studies have shown that antihypertensive or lipid-lowering therapy can decrease A*β* deposition in the brain [69, 70]. Considering the mechanism of hypertension and hyperlipidemia on cognitive impairment mentioned above, we propose a hypothesis that antihypertensive therapy and lipid-lowering may have a synergistic effect on reducing A*β* deposition. In addition, some researchers have proposed that blood pressure control and significant lipid lowering in patients with cognitive decline not only may improve disease control but also could potentially slow the disease progression [71].

### 4.2. Combined Therapy versus Antihypertensive/Lipid-Lowering Therapy Alone

An article, which was published in Neurology in 2019 regarding elderly patients with moderate cardiovascular disease risk, showed that combined antihypertensive and lipid-lowering therapy has no effect on cognitive function decline [72]. It is important to note that Rosuvastatin did not show impairment of cognitive function in the HOPE-3 study, which is different from previous concerns that statins may cause memory loss. At the same time, it is suggested that patients with higher baseline BP and LDL levels have more benefit trends, and long-term blood pressure control may reduce the incidence of cognitive decline. However, the two inferences mentioned above need more clinical researches to confirm.

### 4.3. Which Patients Can Benefit from Combined Antihypertensive and Lipid-Lowering Therapy?

Because of the controversy in the study, it is necessary to comprehensively consider some factors, such as age, baseline blood pressure, blood lipid and glucose levels, baseline cardiovascular risk, follow-up time, cognitive impairment at the time of enrollment, and the target value of blood pressure and lipid reduction. Gottesman et al. proposed an increasing number of midlife vascular risk factors that were significantly associated with elevated amyloid standardized uptake value ratios, which was not significant for late-life risk factors [7]. The main results from the PODCAST randomized controlled trial showed that, in patients with recent stroke and normal cognition, intensive BP and lipid lowering were feasible and safe but did not alter cognition over two years [67]. The small enrolled number, advanced age, and poststroke status may account for these differences.

Therefore, we infer that people in their midlife may benefit from combined antihypertensive and lipid-lowering therapy. The FINGER study confirmed that, through comprehensive intervention including combined antihypertensive and lipid-lowering therapy, other vascular risk factors control, cognitive training, exercise, and diet, among others, can significantly reduce the risk of dementia after 2 years of follow-up [73]. This suggests that patients with mid-term intervention can benefit in their old age.

Besides early intervention, treatment compliance is also a key factor for the efficacy of combined antihypertensive and lipid-lowering therapy, especially for the elderly who have experienced MCI and memory and cognitive decline and also take medication regularly [74]. At the same time, elderly patients often suffer from a variety of diseases, and multiple drugs have become their routine. At present, the continuous emergence of single-pill compound preparations is lifting the burden of “double high” (hypertension and hyperlipidemia) patients, for example, a single-pill combination of amlodipine and atorvastatin, and a two-pronged combination of antihypertensive and lipid-lowering drugs [75, 76].

## 5. Prospect

The combination of antihypertensive and lipid-lowering therapy attracts much more attention in clinical research although there are still some controversies regarding the effects of antihypertensive and lipid-lowering therapy on cognitive function. Hypertension and hyperlipidemia may lead to the accumulation of APP and A*β* through several common mechanisms, including hypoperfusion, ischemia and hypoxia, and blood-brain barrier dysfunction. It is currently believed that hypertension and elevated blood cholesterol levels in middle age may be related to cognitive impairment or dementia in old age. Actively controlling potential risk factors from middle age may be important for AD prevention. Intensive antihypertensive or lipid-lowering therapy may be better than standard antihypertensive or lipid-lowering therapy, but further tests are needed to confirm its effect on cognitive function.

Most researches have shown that there are multiple factors influencing the effects of antihypertensive and lipid-lowering therapy on the cognitive function of hypertension and hyperlipidemia patients, such as age, baseline BP, cholesterol and blood glucose levels, baseline cardiovascular risk, follow-up time, cognitive impairment at the time of enrollment, target value of blood pressure reduction and lipid lowering, and treatment compliance. The early combination of antihypertensive and lipid-lowering therapies can improve cognitive function as early as possible, especially in patients with better treatment compliance. Further researches are required for better clinical practice guidance. At the same time, elderly patients often suffer from multiple diseases, and multiple drugs have become their routine treatment. In the future, further researches are needed to prove the effects of combination of antihypertensive and lipid-lowering therapies on the improvement of cognitive function. In addition, the development of new single-pill drugs that can comanage multiple risk factors such as hypertension and dyslipidemia will bring more benefits to elderly patients.

## Figures and Tables

**Table 1:** Studies into the effect of antihypertensive therapy on cognitive function.

Title	Research design	Age at baseline	Intervention	Duration/follow-up	Main results	Number of participants
Association between blood pressure and Alzheimer disease measured up to 27 years prior to diagnosis: the HUNT study	Large, population-based health study	NF	None	27 years	An inverse association between dementia and systolic blood pressure (BP) in individuals over the age of 60 years.	24,638
Low diastolic pressure and risk of dementia in very old people: a longitudinal study	Dementia-free cohort	≥81	None	3 years	Low diastolic pressure predicts the risk of dementia among very old people.	422
The role of cardiovascular risk factors and stroke in familial Alzheimer disease	Longitudinal study of families with multiple members affected with LOAD	77.0 ± 9	None	2003 to 2015	Hypertension was associated with decreased LOAD risk while type 2 diabetes and heart disease were not. A history of stroke conferred >2-fold increased risk for LOAD.	6553
Nilvadipine in mild-to-moderate Alzheimer disease: a randomized controlled trial	Large-scale phase III investigator-driven clinical trial	>50	Placebo or nilvadipine	18 months	It does not suggest any benefit of nilvadipine as a treatment in a population spanning mild-to-moderate AD.	511
15-year longitudinal study of blood pressure and dementia	Longitudinal population study	70	None	15 years	Subjects who developed dementia at 79 to 85 years of age had significantly higher blood pressure 15 years earlier.	382
Brain aging in very old men with type 2 diabetes: the Honolulu-Asia aging study	Longitudinal population study	81.6 ± 5.0	None	1965 to 1996	Older individuals with type 2 diabetes have an elevated risk for vascular brain damage and neurodegenerative changes.	3,734
Brain aging in very old men with type 2 diabetes: the Honolulu-Asia aging study	Double-blind, placebo-controlled trial of antihypertensives in patients with an untreated SBP of 160–199 mmHg	≥80	Placebo or active treatment	2.2 years	Untreated hypertension was significantly associated with hippocampal atrophy, midlife cognitive decline, AD, and vascular dementia.	3845
Prevention of dementia in randomized double-blind placebo-controlled systolic hypertension in Europe (Syst-Eur) trial	Double-blind placebo-controlled systolic hypertension in Europe (Syst-Eur) trial		Placebo or active treatment	2 years	A wider pulse pressure may indicate an increased risk for dementia, and it was also found that active treatment may change the shape of the relationship between DBP and dementia.	2418
Effects of valsartan compared with enalapril on blood pressure and cognitive function in elderly patients with essential hypertension	Prospective, randomized, open-label, blinded-endpoint study	61 to 80	Valsartan or enalapril	16 weeks	Valsartan (160 mg) is more effective than enalapril (20 mg.) in reducing BP and improves some of the components of cognitive function, particularly episodic memory.	144
Lower dementia risk with different classes of antihypertensive medication in older patients	Randomized controlled trial	74.4 ± 2.5	Different antihypertensive medications	6 to 8 years	Both calcium channel blockers (CCBs) and ARBs are independently associated with a decreased risk of dementia in older people.	1951
Patterns of antihypertensive and statin adherence prior to dementia: findings from the adult changes in thought study	Population-based cohort study	≥65	None	NF	Antihypertensive adherence is an important factor that affects the odds of dementia.	4368
Effect of intensive vs standard blood pressure control on probable dementia: a randomized clinical trial	Randomized clinical trial	≥50	SBP goal of either <120 mm Hg or <140 mm Hg	2010 to 2015	Intensive SBP control can prevent cognitive impairment.	9361
Cognitive function and brain structure in persons with type 2 diabetes mellitus after intensive lowering of blood pressure and lipid levels: a randomized clinical trial	Randomized clinical trial	62	SBP goal of either <120 mm Hg or <140 mm Hg	40 months	A significant cognitive decline was observed after 40 months of intensive BP control in patients with type 2 diabetes.	2977

**Table 2:** Studies into the effect of lipid-lowering therapy on cognitive function.

Title	Research design	Age at baseline	Intervention	Duration/follow-up	Main results	Number of participants
Use of lipid-lowering agents, indication bias, and the risk of dementia in community-dwelling elderly people	A cohort study of lipid-lowering agents (LLA) use and a case-control study of dementia in relation to LLA use	≥65	None	NF	In those younger than 80 years, the usage of lipid-lowering agents was associated with a lower risk of dementia and AD.	2305
Decreased prevalence of Alzheimer disease associated with 3-hydroxy-3-methylglutaryl coenzyme A reductase inhibitors	Cross-sectional study	≥60	None	1996–1998	The prevalence of probable AD in the cohort taking statins was 60–73% lower than the total patient population or compared with patients taking other medications typically used in the treatment of hypertension or cardiovascular disease.	57104
Statins and the risk of dementia	Nested case-control study	≥50	None	NF	Individuals (≥50 years) who were prescribed statins had a substantially lower risk of developing dementia, independent of the presence or absence of untreated hyperlipidemia, or exposure to nonstatin LLAs.	1364
Statins are associated with a reduced risk of Alzheimer disease regardless of lipophilicity: the Rotterdam study	Prospective, population-based Rotterdam study	≥55	None	1990–1993 to 2005	In the general population, the use of statins, but not of nonstatin cholesterol-lowering drugs, was associated with a lower risk of AD compared with the absence of cholesterol-lowering drug usage.	6992
Statin use and the risk of incident dementia: the cardiovascular health study	Cohort study	≥65	None	NF	Statin therapy was not associated with a decreased risk of dementia.	2798
The association of statin use and statin type and cognitive performance: analysis of the reasons for geographic and racial differences in stroke (REGARDS) study	Cross-sectional study	≥45	None	2003–2008	Statin use and type were marginally associated with cognitive impairment. After adjusting for known variables that affect cognition, no association was observed.	24595
The 32-year relationship between cholesterol and dementia from midlife to late life	Prospective population study	38–60	None	32 years	Midlife cholesterol level was not associated with an increased risk of AD.	1462
Intensive versus guideline blood pressure and lipid lowering in patients with previous stroke: main results from the pilot “prevention of decline in cognition after stroke trial” (PODCAST) randomized controlled trial	Randomized clinical trial	74.0 ± 6.8	Intensive (target LDL-cholesterol <1.3 mmol/l) or guideline (target LDL-c <3.0 mmol/l) lipid lowering	2 years	Intensive lipid-lowering therapy was significantly associated with improved scores for ACE-R at 6 months, trail making A, modified Rankin scale, and Euro-Qol visual analogue scale.	83
Improved neurocognitive functions correlate with reduced inflammatory burden in atrial fibrillation patients treated with intensive cholesterol-lowering therapy	Randomized clinical trial	74.5 ± 4.2 (treatment)/73.5 (Pablo)	Atorvastatin (40 mg) and ezetimibe (10 mg) or double placebo	1 year	Intensive lipid-lowering treatment can modify the deterioration of neurocognitive function and the loss of volume in certain cerebral areas in older patients with arterial fibrillation.	34
Patterns of antihypertensive and statin adherence prior to dementia: findings from the adult changes in thought study	Population-based cohort study	≥65	None	NF	No association was detected between statin adherence and dementia.	4368
